# Evaluation of mass drug administration in the program to control imported lymphatic filariasis in Thailand

**DOI:** 10.1186/s12889-015-2325-x

**Published:** 2015-09-28

**Authors:** Tanaporn Toothong, Mathuros Tipayamongkholgul, Nawarat Suwannapong, Saravudh Suvannadabba

**Affiliations:** Department of Epidemiology, Faculty of Public Health, Mahidol University, 420/1 Rajvithi Road, Ratchathewi, Bangkok, 10400 Thailand; Department of Disease Control, Ministry of Public Health, Nonthaburi, 11000 Thailand; Department of Public Health Administration, Faculty of Public Health, Mahidol University, 420/1 Rajvithi Road, Ratchathewi, Bangkok, 10400 Thailand

**Keywords:** Imported filariasis, Undocumented immigrant, Control program, Metropolitan area

## Abstract

**Background:**

Migration plays a major role in the emergence and resurgence of lymphatic filariasis (LF) in many countries. Because of the high prevalence of Imported Bancroftian Filariasis (IBF) caused by nocturnally periodic *Wuchereria bancrofti* and the intensive movement of immigrant workers from endemic areas, Thailand has implemented two doses of 6 mg/kg diethylcarbamazine (DEC) with interval of 6 months to prevent IBF. In areas where immigrants are very mobile, the administration of DEC may be compromised. This study aimed to evaluate DEC administration and its barriers in such areas.

**Methods:**

A cross-sectional study with two-stage stratified cluster sampling was conducted. We selected Myanmar immigrants aged >18 years from factory and fishery areas of Samut Sakhon Province for interview with a structured questionnaire. We also interviewed health personnel regarding the functions of the LF program and practice of DEC delivery among immigrants. Associations were measured by multiple logistic regression, at P <0.05.

**Results:**

DEC coverage among the immigrants was 75 %, below the national target. All had received DEC only once during health examinations at general hospitals for work permit renewals. None of the health centers in each community provided DEC. Significant barriers to DEC access included being undocumented (adjusted OR = 74.23; 95 % CI = 26.32–209.34), unemployed (adjusted OR = 5.09; 95 % CI = 3.39–7.64), daily employed (adjusted OR = 4.33; 95 % CI = 2.91–6.46), short-term immigrant (adjusted OR = 1.62; 95 % CI = 1.04–2.52) and living in a fishery area (adjusted OR = 1.57; 95 % CI = 1.04–2.52). Incorrect perceptions about the side-effects of DEC also obstructed DEC access for Myanmar immigrants. All positive LF antigenic immigrants reported visiting and emigrating from LF-endemic areas.

**Conclusion:**

Hospital-based DEC administration was an inappropriate approach to DEC delivery in areas with highly mobile Myanmar immigrants. Incorporating health-center personnel in DEC delivery twice yearly and improving the perceptions of DEC side effects would likely increase DEC coverage among Myanmar immigrants.

## Background

Thailand is a low-endemic area for lymphatic filariasis (LF). LF cases have only been reported among Thai residents in one southern province, and the country is in the process of verifying the elimination of the disease [1]. However, the high degree of immigrant movement from LF-endemic countries to industrial areas of Thailand, together with an existing potential vector (*Culex quinquefasciatus*)*,* may result in the emergence of LF in these areas [2–6]. High prevalence of Imported Bancroftian Filariasis (IBF) caused by the nocturnally periodic *Wuchereria bancrofti* among Myanmar immigrants over recent decades [4, 7] has spurred the Thai Ministry of Public Health (MoPH) to implement a countrywide biannual treatment program using 6 mg/kg of diethylcarbamazine (DEC) for all Myanmar immigrants to prevent IBF transmission [8]. Multiple doses of DEC have shown long-term efficacy with microfilariae (MF) [9–14] and macrofilariae [11]. Due to low cost and minimal toxicity [13, 14], two doses DEC with interval of 6 months is more feasible and cost-effective for preventing IBF transmission among highly mobile populations [9–14].

To reduce the sources of LF infection effectively, DEC coverage among eligible immigrants must be >90 %, while the prevalence of MF antigen must be <1 % [8]. The MoPH uses two strategies to deliver DEC biannually to immigrants: through general hospitals during work permit renewal, and through health center outreach to communities [15]. Metropolitan areas commonly have high levels of population mobility and high numbers of undocumented immigrants, which may compromise DEC administration by cumulative delayed or missed doses [12]. Moreover, convenient transportation may enable Myanmar immigrants to commute from the Myanmar border to metropolitan areas and serve as active sources of LF infection.

Therefore, this study aimed to evaluate biannual DEC administration and to identify barriers to DEC access among Myanmar immigrants in a metropolitan area. The findings of this study may provide insights regarding the situation of IBF control in areas where Myanmar immigrants are highly mobile and improve the effectiveness of DEC administration and IBF prevention & control programs in metropolitan areas.

## Methods

### Study design, subjects and description of the study site

**Fig. 1:**
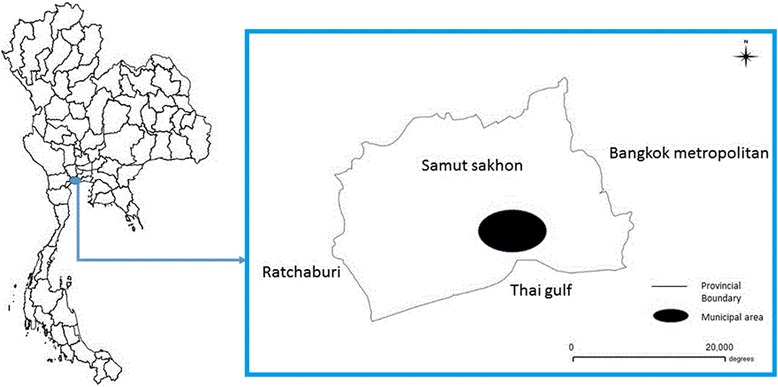
Map of Thailand with study area: Samut Sakhon Province. (Solid line presented provincial boundary and black color oval presented municipal area)

A cross-sectional study, using both quantitative and qualitative methods, was conducted in a metropolitan area with large seafood-processing and fishing industries, in Samut Sakhon Province, Thailand (Fig. 1). Samut Sakhon is located 30 km south of Bangkok and north of the gulf of Thailand. This area comprises the second highest number of Myanmar immigrants, following Bangkok. Two-stage stratified cluster sampling was used to select six Myanmar communities from factory and fishery areas. Two Myanmar immigrants aged >18 years were selected from each household in the sample area. The estimated sample size was calculated by single proportion estimation, with alpha level 0.05, DEC coverage proportion 52.0 % [15], precision error 5 %, and design effect two. The study sample required a minimum of 767 Myanmar immigrants. All local health personnel responsible for LF-control programs in the selected communities were also recruited for interview.

### Data collection and analysis

This study used semi-structured questionnaires to interview Myanmar immigrants and dialog guidelines to interview local health personnel responsible for the LF program. Four Myanmar translators, fluent in Thai and Burmese, were trained to translate during the dialog and interview. Local health personnel were asked questions related to the functions of the LF program and practice of DEC delivery among immigrants. Percentage and mean (SD) were used to describe the data. Chi-square test and multiple logistic regression were used to calculate odds ratios (OR) and identify barriers to DEC access. Significance was set at 5 % of alpha, with 95 % confidence level. The research protocol was approved by the Ethics Committee of the Faculty of Public Health, Mahidol University (COA. No. MUPH 2013–133).

## Results

In total, 939 Myanmar immigrants were included in the study and 75 % of the study sample was received DEC only once annually. When asked when and where they obtained their drugs, all responded, “During physical examination for work permit renewal at the hospital”. The proportion of DEC access reported between documented immigrants (81.7 %) and undocumented immigrants significantly differed (5.1 %) (*P* <0.001) (Table 1). Immigrants older than 50 years reported the lowest proportion of DEC (*P* = 0.038). The proportion of DEC accessibility significantly differed between married, single and widowed (*P* = 0.034). Of those employed monthly, 88.3 % had accessed DEC compared with 64.4 % of unemployed (*P* < 0.001). A lower reported DEC intake was observed among immigrants who recently arrived in Thailand (≤12 months) than immigrants affirming longer lengths of stay (*P* = 0.009). Finally, respondents living in fishing areas tended to access DEC at a significantly lower proportion (69.2 %) than those living in factory areas (81.2 %). Sex and educational status were not significantly associated with different proportions of DEC accessibility (Table 1).

**Table 1:** Accessibility of DEC by individual characteristics (*n* = 939)

Variable	Total	Accessibility of DEC				*P*
		Yes (*n* = 707)		No (*n* = 232)		
		N	%	N	%	
Documented						<0.001
Yes	860	703	81.7	157	18.3	
No	79	4	5.1	75	94.9	
Sex						0.351
Female	542	402	74.2	140	25.8	
Male	397	305	76.8	92	23.2	
Age group (years)						0.038
<21	191	150	78.5	41	21.5	
21–30	338	243	71.9	95	28.1	
31–40	237	191	80.6	46	19.4	
41–50	116	86	74.1	30	25.9	
>50	57	37	64.9	20	35.1	
Marital status						0.034
Married	652	478	73.3	174	26.7	
Other	287	229	79.8	58	20.2	
Occupation						<0.001
Unemployed	267	172	64.4	95	35.6	
Employed monthly	453	400	88.3	53	11.7	
Employed daily	219	135	61.6	84	38.4	
Education status						0.405
None	225	175	77.8	50	22.2	
Primary school	481	358	74.4	123	25.6	
Secondary school	215	163	75.8	52	24.2	
University	18	11	61.1	7	38.9	
Length of stay in community (months)						
≤12	169	112	66.3	57	33.7	0.009
13–36	221	178	80.5	43	19.5	
37–60	157	123	78.3	34	21.7	
>60	392	294	75.0	98	25.0	
Living zone						<0.001
Factory area	478	388	81.2	90	18.8	
Fishery area	461	319	69.2	142	30.8	

*P* from Chi-square test; significant level at 0.05

Five interviewed health personnel reported that two general hospitals were responsible for providing DEC to all immigrants during physical examination at the hospitals. In addition, they had not been involved in DEC administration for longer than ten years. None of the health personnel had delivered a second dose to Myanmar immigrants.

Multiple logistic regression analysis was used to examine obstacles to DEC access among the Myanmar immigrants. The results showed that undocumented immigrants were 74.23 times more likely to have obstacles to DEC access than documented immigrants (95%CI = 26.32–209.34), while unemployed and daily employed participants had substantially greater obstacles to DEC access compared with participants employed monthly (OR = 5.09; 95%CI = 3.39–7.64 and OR = 4.33; 95%CI = 2.91–5.46, respectively). Short term Myanmar immigrants, and those living in fishery areas were more likely to suffer obstacles to DEC access than other groups (OR = 1.62; 95 % CI = 1.04–2.52 and OR = 1.57; 95%CI = 1.01–2.26, respectively) after adjusting for age, sex, educational status and marital status (Table 2).

**Table 2:** Factors associated with lack of access to DEC

Variable	Inaccessibility of DEC			*P*
	Crude	Adjusted	95 % CI of OR	
	OR	OR	Lower-upper	
Documented				
Yes	1	1		
No	83.96	74.23	26.32–209.34	<0.001
Occupation				
Employed monthly	1	1		
Unemployed	4.70	5.09	3.39–7.64	<0.001
Employed daily	4.17	4.33	2.91–6.46	<0.001
Length of stay in community (months)				
≤12	1.53	1.62	1.04–2.52	0.032
13–36	0.73	0.78	0.78–0.50	0.262
37–60	0.83	0.87	0.54–1.40	0.560
>60	1	1		
Living zone				
Factory areas	1	1		
Fishery areas	1.92	1.57	1.09–2.26	0.015

*P* from Chi-square test; significant level at 0.05, OR is Odds Ratio from logistic regression, Adjusted OR from multiple logistic regression, adjusted for age group, sex, educational status and marital status

## Discussion

The overall coverage of DEC among immigrant was 75 %. The proportion of documented and undocumented immigrants who have received DEC equaled 81.7 % and 5.1 %, respectively. A similar level of coverage was reported in southern Thailand [12, 15, 16].

Low DEC accessibility was also found in the fishery areas and was likely related to the higher proportion of undocumented immigrants (13 %), compared with factory areas (3.9 %). The low DEC coverage might have resulted from DEC-administration practices, namely, DEC was provided by two general hospitals during health examinations for the renewal of work permits. Even though health centers were designated one of the two strategies to deliver bi-annual DEC to immigrants according to national guidelines [8, 15], no health center reported DEC administration. This fact indicated a lack of DEC access among undocumented immigrants who mostly used health center services.

Undocumented immigrants commonly accessed healthcare services at general hospitals less because of their illegal status [17–19]. Additionally, undocumented immigrants living in fishery areas mostly worked on fishing vessels, and frequently went to sea for months at a time resulting in untreated DEC. Undocumented immigrants receiving only a single annual DEC treatment can gain partial protection against microfilariae, but not macrofilariae [9, 10].

The suboptimal DEC administration may not interrupt IBF transmission in areas with high population movement from endemic areas. Local health personnel, responsible for the LF program, face a great challenge. They must disseminate health information to undocumented immigrants and their employers that multiple DEC treatment is a key factor to prevent LF transmission and benefits the health of their employees. The high movement of Myanmar immigrants has suggested the need to strengthen the surveillance system for LF at border checkpoints and immigration stations regarding the national policy for border health prevention and control [20].

No immigrant who received the first round of DEC received a second dose. The Thai helminthiasis-control DOTS policy requires Myanmar immigrants to ingest a single dose of 300 mg DEC and 400 mg albendazole in front of the nurse during physical examination for work permit renewal. This drug combination completely eradicates both microfilariae and macrofilariae [9, 10, 21]. Due to the policy, we expected 100 % DEC coverage among documented immigrants. Apparently, DEC intake during work permit renewal has not been strictly enforced because the reported DEC coverage among documented immigrants did not reach expectations. Therefore, the national LF program should strengthen monitoring and evaluation at general hospitals.

Factors associated with DEC inaccessibility included being undocumented, employed daily, unemployed, short-term immigration and living in a fishery area. Being an undocumented immigrant was strongly associated with impeded DEC access. This finding may be related to undocumented immigrants’ fear of accessing healthcare services because of their undocumented status [18–20]. A significant association was found between being unemployed or employed daily and impeded DEC access, consistent with previous studies conducted in southern Thailand [12, 15, 16]. This can be explained by the socioeconomic status of these groups of immigrants. Unemployed immigrants and immigrants employed daily had low education levels and limited incomes. They barely visited hospitals because of potential lost income and time [20]. The low DEC coverage among short term immigrants and those living in fishery areas confirmed the results of other studies [12, 16]. They were highly mobile, resulting in reduced access to healthcare services [12, 16, 18]. Moreover, negative perceptions about the side effects of DEC were reported among the Myanmar immigrants, which created another barrier to DEC administration in this area.

This study found that hospital-based administration of DEC is inappropriate in areas with high proportions of undocumented immigrants and highly mobile populations. Thailand has a well-developed healthcare infrastructure. At lower levels of health services delivery such as health centers personnel work actively on disease prevention and control and generally know the community context rather well. Health-center personnel can identify the homes of all community members and easily deliver DEC to undocumented immigrants [22–25]. In Thailand, almost all undocumented immigrants are daily employed workers living in fishery areas. Therefore, local health personnel can target active DEC delivery in this group. Moreover, health-center personnel work closely with community leaders and members, and can improve the perceptions and understanding of DEC administration among Myanmar immigrants and their employers. Health personnel can reduce fears about illegal status among undocumented immigrants because they normally live in the area and are familiar with community members, such as their employers [23, 25]. As a result, including health centers in DEC administration should improve both accessibility and coverage. In addition, the national LF program should strengthen close surveillance and monitoring of LF programs in highly mobile populations.

## Conclusion

Our study revealed that the IBF component of the Thai National Program to Eliminate Lymphatic Filariasis in study site did not achieve the desired goals as hospital-based DEC administration did not reach undocumented and short term Myanmar immigrants. Moreover, such a strategy was unable to provide DEC to Myanmar immigrants bi-annually, according to policy. To increase DEC coverage, health center-based DEC administration is suggested as a more effective way of reaching undocumented immigrants.
